# Supplementary material for: Mutations in PCYT2 disrupt etherlipid biosynthesis and cause a complex hereditary spastic paraplegia
Source: Brain. 2019 Oct 22;142(11):3382–97. doi: 10.1093/brain/awz291 (PMC6821184; doi:10.1093/brain/awz291)
Supplement: awz291_Supplementary_Data [file awz291_supplementary_data.zip › awz291-Suppl_data/Supplementary_Data4.zip!/Supplementary information 4 - plasma lipidomics.zip!/Plasma lipidomics/Multiple comparison/PLS-DA.pdf]

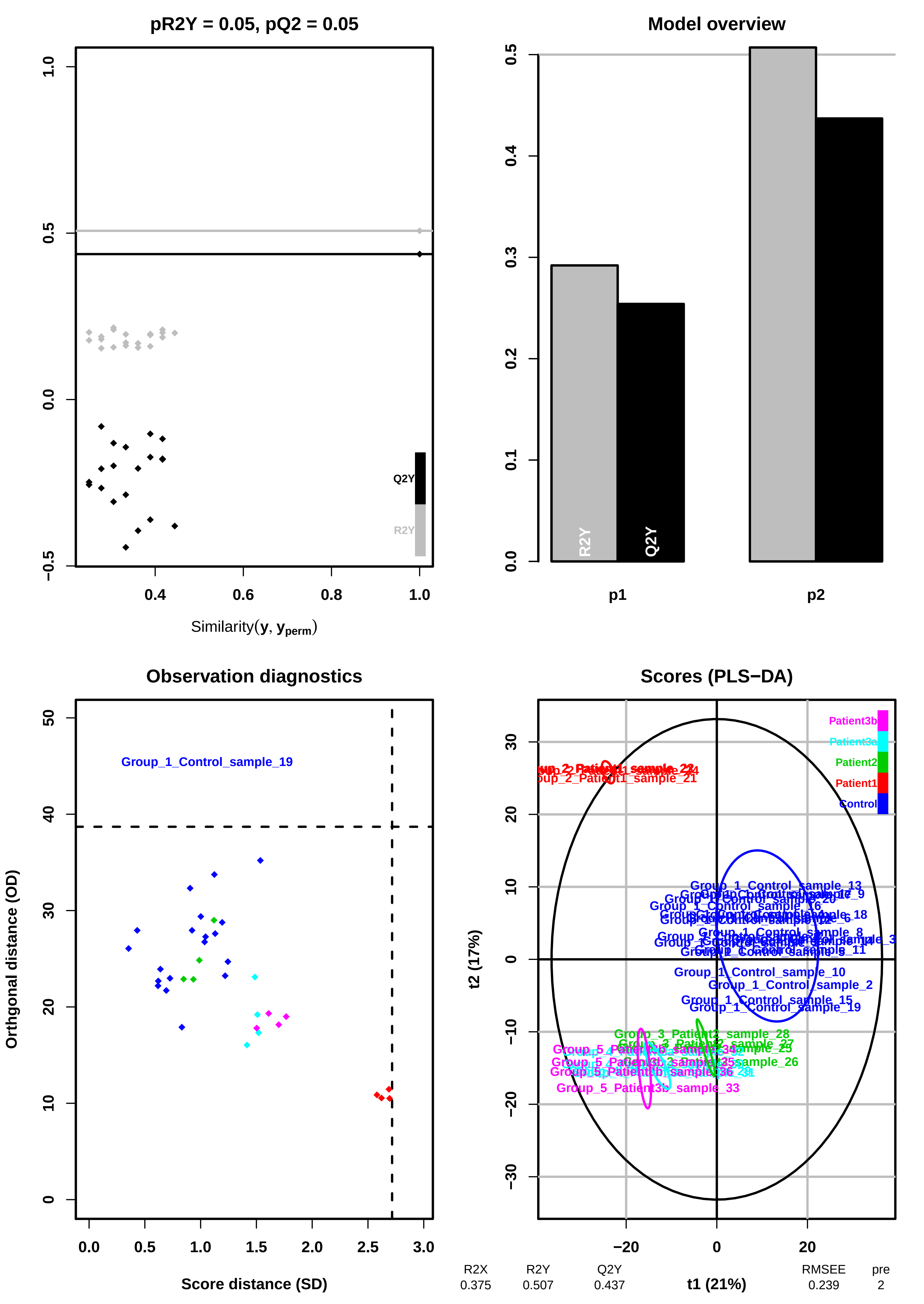

pR2Y = 0.05, pQ2 = 0.05
Similarity(y, y_perm)
Q2Y
R2Y
Model overview
p1
p2
Observation diagnostics
Group_1_Control_sample_19
Orthgonal distance (OD)
Score distance (SD)
Scores (PLS−DA)
Patient3b
Patient3a
Patient2
Patient1
Control
Group_1_Control_sample_13
Group_1_Control_sample_16
Group_1_Control_sample_8
Group_1_Control_sample_10
Group_1_Control_sample_2
Group_1_Control_sample_15
Group_1_Control_sample_19
Group_3_Patient2_sample_28
Group_5_Patient3b_sample_33
t1 (21%)
t2 (17%)
R2X
0.375
R2Y
0.507
Q2Y
0.437
RMSEE
0.239
pre
2
